# Effect of the interaction between alcohol and meat consumption on the hyperlipidaemia risk among elderly individuals: Evidence from Shanghai, China

**DOI:** 10.3389/fnut.2022.982626

**Published:** 2022-10-17

**Authors:** Xiaojing Huang, Hong Hui, Wenqing Zhu, Ning Chen, Yan Wei, Zhaoxin Wang, Jianwei Shi

**Affiliations:** ^1^School of Management, Xuzhou Medical University, Xuzhou, China; ^2^General Department, Shanghai Baoshan District Gucun Town Community Health Service Center, Shanghai, China; ^3^Executive Office, Shanghai Municipal Center for Disease Control and Prevention, Shanghai, China; ^4^School of Public Health, Shanghai Jiaotong University School of Medicine, Shanghai, China; ^5^Key Lab of Health Technology Assessment (National Health Commission), School of Public Health, Fudan University, Shanghai, China; ^6^Health Management Center, First Affiliated Hospital of Hainan Medical University, Hainan, China; ^7^School of Management, Hainan Medical University, Hainan, China; ^8^Department of General Practice, Yangpu Hospital, Tongji University School of Medicine, Shanghai, China; ^9^Department of Social Medicine and Health Management, School of Public Health, Shanghai Jiaotong University School of Medicine, Shanghai, China

**Keywords:** hyperlipidaemia risk, alcohol drinking, meat consumption, marginal effect, interaction effect

## Abstract

**Background:**

Diet and other lifestyle habits may have an increased effect on blood lipids in older people. This study aimed to examine the associations between diet (meat, fish, and egg), alcohol consumption and blood lipids.

**Methods:**

Surveillance data on chronic diseases and their risk factors were collected from Shanghai during 2017–2018. A Kish table was used for sampling 438 older adults, of whom 71 consumed alcohol. Logistic regression was used to test the relationships between diet, alcohol consumption and blood lipid levels in elderly individuals, and the marginal effects (MEs) were estimated.

**Results:**

Dyslipidaemia was more common among drinkers than among nondrinkers (*P* < 0.01). Alcohol consumption was associated with dyslipidaemia (OR = 2.667, *P* < 0.01 for TC; OR = 1.919, *P* < 0.05 for LDL; OR = 3.412, *P* < 0.01 for TG), and consumption of more than 50 g of meat per day showed similar associations (OR = 3.227, *P* < 0.01 for TC; OR = 3.263, *P* < 0.01, for LDL; OR = 2.329, *P* < 0.01 for TG). The MEs of alcohol drinking and excessive meat consumption on the rate of dyslipidaemia were 0.324 for TC (*P* < 0.01), 0.255 for LDL (*P* < 0.05), and 0.174 for TG (*P* < 0.01).

**Discussion:**

The risk of hyperlipidaemia was increased among elderly individuals with excessive meat and alcohol consumption, which also had an interactive effect.

## Introduction

Hyperlipidaemia is defined as increased levels of plasma total cholesterol (TC), low-density lipoprotein-cholesterol (LDL-C), or triglycerides (TGs) with or without decreased levels of high-density lipoprotein cholesterol (HDL-C) (1). LDL-C and HDL-C regulate the amount of cholesterol in the body, and abnormal levels of either can cause an imbalance, so hyperlipidaemia is one of the main risk factors that contributes to atherosclerosis and coronary artery disease (CAD) (2). In China, the number of patients with coronary heart disease (CHD) and stroke-related deaths is expected to increase above the current rate of 3 million deaths annually (3, 4). The overall prevalence of dyslipidaemia among Chinese adults was 40.4% in 2015, which was significantly higher than that in 2002 (18.6%) (5). Hyperlipidaemia has emerged as the major contributor to the increase in CHD mortality (by 77%) in China (6, 7), and it is vital to control high lipid levels to prevent poor cardiovascular outcomes and improve quality of life (QoL) (8–10).

Obesity and a diet high in saturated fats are important modifiable risk factors for hyperlipidaemia (11). Epidemiological studies indicate that a high intake of red meat is associated with an increased risk of dyslipidaemia (12, 13). Red meat consumption was found to be cross-sectionally associated with the occurrence of hypertriglyceridaemia (14). Some randomized controlled studies show that white meat and red meat induce a similar lipid response (15–17). The saturated fatty acid in meat may have been a factor that contributed to this association. However, long-term fish intake was found to be associated with reduced levels of total serum cholesterol and triglycerides in a cohort of elderly people (18–20). Although the impact of eggs on disease has contradictory evidence, 3 large international prospective studies from 50 countries on 6 continents did not find a significant association between egg intake and blood lipids (21).

Some other factors can be regulators or mediators of hyperlipidaemia. Alcohol is an important regulator of blood lipid levels. Studies have found that alcohol consumption is a negative factor, especially when there are other unhealthy lifestyle behaviors, and that excessive alcohol consumption increases the risk of dyslipidaemia (22–24). Some studies suggest that alcohol may have a protective effect. A study in Japan found that among people over 40 years old, the alcohol consumption pattern was inversely associated with the prevalence of dyslipidaemia among women (25). Another study in Brazil reported that runners who drank moderately were less likely to have hyperlipidaemia (26). Obesity and alcohol consumption are mediating variables. A cohort study confirmed that abdominal obesity is related to a high-fat diet and can accelerate the occurrence of dyslipidaemia (27). Physical activity is another regulatory factor. An earlier study showed that physical activity independently reduced LDL-cholesterol levels in a cohort of middle-aged men and women (28).

In China, dietary imbalance has become the main risk factor for chronic diseases (29, 30). According to statistics, the per capita consumption of red meat by Chinese residents in 2018 was 29.5 kg, an increase of 15.23% compared with 25.6 kg in 2014, and the per capita consumption of poultry increased from 8.0 to 9.0 kg, with a growth rate of 12.5% (31). The consumption of livestock and poultry meat has exceeded the standard set by *Dietary Guidelines for Chinese Residents* of 75 g per person per day (27.4 kg per person per year) (32), which is similar to Swedish and Finnish standards (33). In the same period, the consumption of vegetables decreased by 1.1% (31) and was lower than the standard in the *Dietary Guidelines for Chinese Residents*. In 2015, a study in 15 provinces of China showed that the average dietary cholesterol intake of 28% of older adults was higher than 300 mg/day (34). For every 100 mg increase in cholesterol intake among older adults, TC levels increased by 0.0409 mmol/L, and LDL-C levels increased by 0.0280 mmol/L (34). With substantial changes in lifestyles, the duration of physical activity has decreased, and the obesity rate has increased among elderly individuals in China. In addition, the proportion of adult Chinese men who consume alcohol was 64.5%, and that of women was 23.1% in 2015 (29). These trends may lead to the occurrence of dyslipidaemia in older adults.

Shanghai, a metropolitan city, was the first city in China to have a substantial aging population and has the most rapid rate of aging among large cities in China. In 2018, it had 14.6 million registered residents, including an estimated 5.02 million elderly people who were over 60 years old (35). The detection rate of dyslipidaemia in physical examinations of elderly individuals in Shanghai was 40.0~72.5% (36, 37). Shanghai attaches great importance to the dietary health of residents and has distributed books on dietary nutrition, balanced diet quick checklists and other relevant materials throughout the communities. Moreover, many communities have built canteens or provided catering services for elderly individuals. These services not only provide elderly residents with food but also help them eat healthily. However, data about the healthiness of catered meals for elderly individuals are rarely reported.

An increasing number of studies on the correlation between food and chronic disease occurrence and treatment have been carried out, and other studies have reported the results of dietary interventions. Few studies have reported the combined effect of food intake and other living habits on blood lipids in the elderly population in recent years in China. To investigate the influence of these factors on blood lipid levels in the elderly population, this study assessed the relationships of blood lipids with the main diet, alcohol consumption habits and personal characteristics of elderly individuals.

## Methods

### Data source and data screening

The data were from the Regular Monitoring of Chronic Diseases in Yangpu District, a programme of the Shanghai Centers for Disease Control and Prevention, from October 2017 to March 2018. This survey included 1,004 residents from 240 households in all 12 community streets in Yangpu District, Shanghai. Twenty households were randomly selected from each street. A Kish table was used for sampling, and a household survey was conducted. The questionnaire was administered by professional investigators, who input the investigation results into the information collection system at the same time as the interview. In addition to answering the questionnaire, the selected participants received a unique bar code and went to a designated medical institution to undergo a physical examination, which included height, weight, blood pressure, and blood and urine tests. The examination results were recorded in the body measurement table and entered into the database by the investigators. This analysis was approved by the research ethics committee of Tongji University (ref: LL-2016-ZRKX-017).

The survey was divided into three sections: i) basic personal information, including age, sex, education level, marital status, and BMI; ii) personal diet (meat, fish and egg), alcohol consumption (alcohol volume ≥14%) and physical activities; and iii) personal health status, such as body mass index (BMI), fasting glucose, glycosylated hemoglobin, total cholesterol (TC), high-density lipoprotein (HDL), low-density lipoprotein (LDL), triglycerides (TG), urinary protein-to-creatinine ratio, aSBP and aDBP, which were tested at a health institution, as well as a self-reported history of chronic diseases diagnosed by doctors. In part ii, the instrument for collecting the dietary data is the Chinese Diet Balance Index (DBI-16) questionnaire. Due to different diet patterns, we offered four recall methods, including daily diet recalls, 1-week diet recalls, 1-months diet recalls and 1-year diet recalls. In the DBI-16, livestock meat and poultry meat are grouped into one category, and fish meat is in another category. In addition, clinical randomized trials have shown that there is no significant difference in the effects of red meat and white meat on blood lipids (15–17). Therefore, the meat of livestock and poultry were not analyzed separately in this study.

In total, 1,004 people were invited to participate, and 32 people whose physiological and dietary indicators were missing were excluded (the effective response rate was 96.81%). Those who were under 60 years old were also excluded (*n* = 363). Considering that patients with a diagnosis of hypertension, diabetes, or hyperlipidaemia are treated with drugs that may affect blood lipid indicators, 171 patients with these conditions were excluded. Finally, 438 older adults were included, of whom 71 consumed alcohol in the last year.

### Outcome variables

Dyslipidaemia was defined as TC > 5.72 mmol/L, HDL < 0.91 mmol/L, LDL > 3.64 mmol/L or TG > 1.7 mmol/L, according to the Special Group on Prevention and Treatment of Dyslipidaemia of the Editorial Committee of the Chinese Journal of Cardiovascular Diseases.

### Independent variables

The demographic variables were age, sex, education and marital status. In accordance with WHO statistics (38), age was divided into four categories: 60–64, 65–69, 70–74 and ≥75 years old. Education level was divided into primary school or below, junior high school, senior high school, and bachelor's degree or above. BMI was grouped as ≤ 23.9 and >23.9 (39, 40). Among elderly individuals, the consumption of meat (including the meat of livestock and poultry), fish and eggs was more than 50 g per person per day, which is an excessive amount according to the dietary guidelines of Chinese residents (32). The older adults were divided into drinkers and non-drinkers according to whether they consumed alcohol last year. The metabolic equivalents (METs) of physical activities were divided into meeting WHO recommendations (MET-minutes/week ≥ 600) and not meeting WHO recommendations (MET-minutes/week < 600).

### Statistical analysis

Sociodemographic factors, BMI, physical activity, diet and blood lipid levels were compared between the drinking and nondrinking groups using standard descriptive methods, such as frequency distributions and the chi-square test. Logistic regression models were used to test the association between blood lipid levels and the factors of interest. Multiplicative interaction models were used to analyse the effects of the interactions of risk factors on blood lipid levels. The marginal effects using the means were calculated after the estimation of logistic regression models. All statistical tests were conducted using Stata 14 (StataCorp LLC, College Station, TX, USA). A *p*-value < 0.05 was considered indicative of statistical significance. GraphPad Prism 8 (GraphPad Software, San Diego, CA, USA) was used to generate a scatter bar chart of the marginal effects.

## Results

### Sample characteristics and drinking

Table 1 presents the sociodemographic characteristics, BMI, PA and diet according to drinking status. For the overall sample, the average age was 68.17 ± 7.53 years, and 41.1% of participants were men. A total of 68.8% were under the age of 70 years; 86.1% had junior high school and senior high school education; 93.2% were married; and 55% were overweight. The proportion of people who consumed alcohol according to the WHO recommendation was 76.1%, which was higher than that of people who did not consume alcohol (*P* < 0.01). Furthermore, 40.6% of people consumed more than 50 g of meat, 30.6% of people consumed more than 50 g of fish, and 10.3% of people consumed more than 50 g of eggs. A higher proportion of drinkers had excessive meat consumption than those who did not drink (*P* < 0.01).

**Table 1:** Sociodemographic information, BMI, PA and diet of the sample according to drinking status.

**Characteristics**	**Total**		**Drinking**		**No drinking**		***P***
	***n***	**%**	***n***	**%**	***n***	**%**	
Age (years)							0.19
60–64	179	40.9	36	50.7	143	39.0	
65–69	122	27.9	16	22.5	106	28.9	
70–74	61	13.9	6	8.5	55	15.0	
≥75	76	17.4	13	18.3	63	17.2	
Sex							0.13
Male	180	41.1	35	49.3	145	39.5	
Female	258	58.9	36	50.7	222	60.5	
Education							0.58
Primary school or below	53	12.1	6	8.5	47	12.8	
Junior high school	176	40.2	32	45.1	144	39.2	
Senior high school	201	45.9	31	43.7	170	46.3	
Bachelor's degree or above	8	1.8	2	2.8	6	1.6	
Marital status							0.56
Unmarried	30	6.8	6	8.5	24	6.5	
Married	408	93.2	65	91.5	343	93.5	
BMI							0.78
≤ 23.9	197	45.0	33	46.5	164	44.7	
>23.9	241	55.0	38	53.5	203	55.3	
Physical activity (METs)							**< 0.01**
< 600	206	47.0	17	23.9	189	51.5	
≥600	232	53.0	54	76.1	178	48.5	
Meat (g)							**< 0.01**
≤ 50	260	59.4	24	33.8	250	68.1	
>50	178	40.6	47	66.2	117	31.9	
Fish (g)							0.63
≤ 50	304	69.4	51	71.8	253	68.9	
>50	134	30.6	20	28.2	114	31.1	
Egg (g)							0.16
≤ 50	393	89.7	67	94.4	326	88.8	
>50	45	10.3	4	5.6	41	11.2	

Note: The bold values indicate statistical significance.

The different distributions of blood lipid levels by drinking status are displayed in Table 2. The proportions of abnormal LDL and TG levels were both more than 30%. The proportion of abnormal HDL levels was the lowest, which was 8.0%. A higher percentage of drinkers than non-drinkers had abnormal levels of lipids indicators other than HDL (*P* < 0.01). The percentage of abnormal HDL values among non-drinkers was higher than that among drinkers (*P* < 0.01).

**Table 2:** The blood lipid distributions according to drinking status.

**Outcome**	**Total**		**Drinking**		**No drinking**		***P***
	***n***	**%**	***n***	**%**	***n***	**%**	
TC							**< 0.01**
≤ 5.72	323	73.7	38	53.5	285	77.7	
>5.72	115	26.3	33	46.5	82	22.3	
HDL							**< 0.01**
< 0.91	35	8.0	59	83.1	344	93.7	
≥0.91	403	92.0	12	16.9	23	6.3	
LDL							**< 0.01**
≤ 3.64	292	66.7	37	52.1	255	69.5	
>3.64	146	33.3	34	47.9	112	30.5	
TG							**< 0.01**
≤ 1.7	287	65.5	27	38.0	260	70.8	
>1.7	151	34.5	44	62.0	107	29.2	

Note: The bold values indicate statistical significance.

### Multiple logistic regression analysis of blood lipids

Table 3 presents the associations between blood lipid levels and other variables. Drinkers had a higher risk of dyslipidaemia than those who did not drink (OR = 2.667, *P* < 0.01 for TC; OR = 1.919, *P* < 0.05 for LDL; OR = 3.412, *P* < 0.01 for TG). Older adults who consume more than 50 g of meat per day had a higher risk of dyslipidaemia than those who consume < 50 g (OR = 3.227, *P* < 0.01 for TC; OR = 3.263, *P* < 0.01 for LDL; OR = 2.329, *P* < 0.01 for TG). The logistic regression on TC showed that older adults aged 65–69 had a lower risk of abnormal TC than those aged 60–64 (OR = 0.501, *P* < 0.05) and that women were at higher risk than men (OR = 1.770, *P* < 0.05). The results of the logistic regression on HDL were not statistically significant (*P* > 0.05). The logistic regression on LDL showed that overweight and inactivity (METs < 600) were associated with dyslipidaemia (OR = 1.599, *P* < 0.05 for overweight; OR = 1.739, *P* < 0.05 for inactivity). Finally, the logistic regression on TG showed that excessive fish intake was associated with higher TG levels (OR = 1.606, *P* < 0.05).

**Table 3:** Multiple logistic regression analysis of 4 blood lipid indicators.

	**TC**		**HDL**		**LDL**		**TG**	
	**OR**	***P***	**OR**	***P***	**OR**	***P***	**OR**	***P***
Age (years)								
60–64	Reference
65–69	0.501	**< 0.05**	1.106	0.83	0.715	0.22	0.844	0.53
70–74	0.840	0.64	0.682	0.57	0.905	0.78	0.584	0.14
≥75	0.896	0.75	1.660	0.29	0.562	0.09	0.840	0.58
Sex								
Male	Reference
Female	1.770	**< 0.05**	0.773	0.49	1.545	0.06	1.177	0.47
Education								
Primary school or below	Reference
Junior high school	1.262	0.56	2.351	0.29	0.771	0.48	1.019	0.96
Senior high school	0.931	0.86	1.984	0.39	0.488	0.05	0.615	0.20
Bachelor's degree or above	0.495	0.54	2.403	0.53	0.208	0.17	0.890	0.89
Marital status								
Unmarried	Reference
Married	1.574	0.37	2.770	0.34	1.950	0.16	2.682	0.05
BMI								
≤ 23.9	Reference
>23.9	1.199	0.45	1.647	0.19	1.599	**< 0.05**	1.520	0.06
Physical activity (METs)								
≥600	Reference
< 600	1.531	0.10	1.014	0.97	1.739	**< 0.05**	1.002	0.99
Alcohol consumption								
No	Reference
Yes	2.667	**< 0.01**	2.993	0.01	1.919	**< 0.05**	3.412	**< 0.01**
Meat (g)								
≤ 50	Reference
>50	3.227	**< 0.01**	0.906	0.80	3.263	**< 0.01**	2.329	**< 0.01**
Fish (g)								
≤ 50	Reference
>50	1.448	0.14	1.007	0.99	1.208	0.43	1.606	**< 0.05**
Egg (g)								
≤ 50	Reference
>50	1.484	0.30	0.901	0.87	1.495	0.25	1.459	0.29

Note: The bold values indicate statistical significance.

As shown in Table 4, alcohol drinking and excessive meat consumption had a multiplicative interaction effect on TC, LDL, and TG levels (*P* < 0.01). Older adults who did not drink but consumed an excessive amount of meat had a higher risk of dyslipidaemia than those who consumed less meat (OR = 3.144, *P* < 0.01 for TC; OR = 3.180, *P* < 0.01 for LDL; OR = 2.525, *P* < 0.01 for TG). Older drinkers who consumed an excessive amount of meat had a higher risk of dyslipidaemia than those who consumed less meat (OR = 7.304, *P* < 0.01 for TC; OR = 4.677, *P* < 0.01 for LDL; OR = 7.631, *P* < 0.01 for TG). Drinkers who consumed < 50 g of meat per day had a higher risk of higher TG levels than non-drinkers who consumed < 50 g of meat (OR = 2.739, *P* < 0.05).

**Table 4:** Multiplicative interaction effects in the logistic regression models.

**Multiplicative interaction**		**TC**		**HDL**		**LDL**		**TG**	
		**OR**	***P***	**OR**	***P***	**OR**	***P***	**OR**	***P***
Drinking × meat		4.648	**< 0.01**	2.269	0.07	3.086	**< 0.01**	5.453	**< 0.01**
Drinking	Meat								
No	≤ 50	1.000		1.000		1.000		1.000	
No	>50	3.144	**< 0.01**	0.931	0.88	3.180	**< 0.01**	2.252	**< 0.01**
Yes	≤ 50	1.803	0.24	3.849	0.02	1.426	0.45	2.739	**< 0.05**
Yes	>50	7.304	**< 0.01**	2.559	0.05	4.677	**< 0.01**	7.631	**< 0.01**

Note: The bold values indicate statistical significance.

### Marginal effects

**Figure 1:**
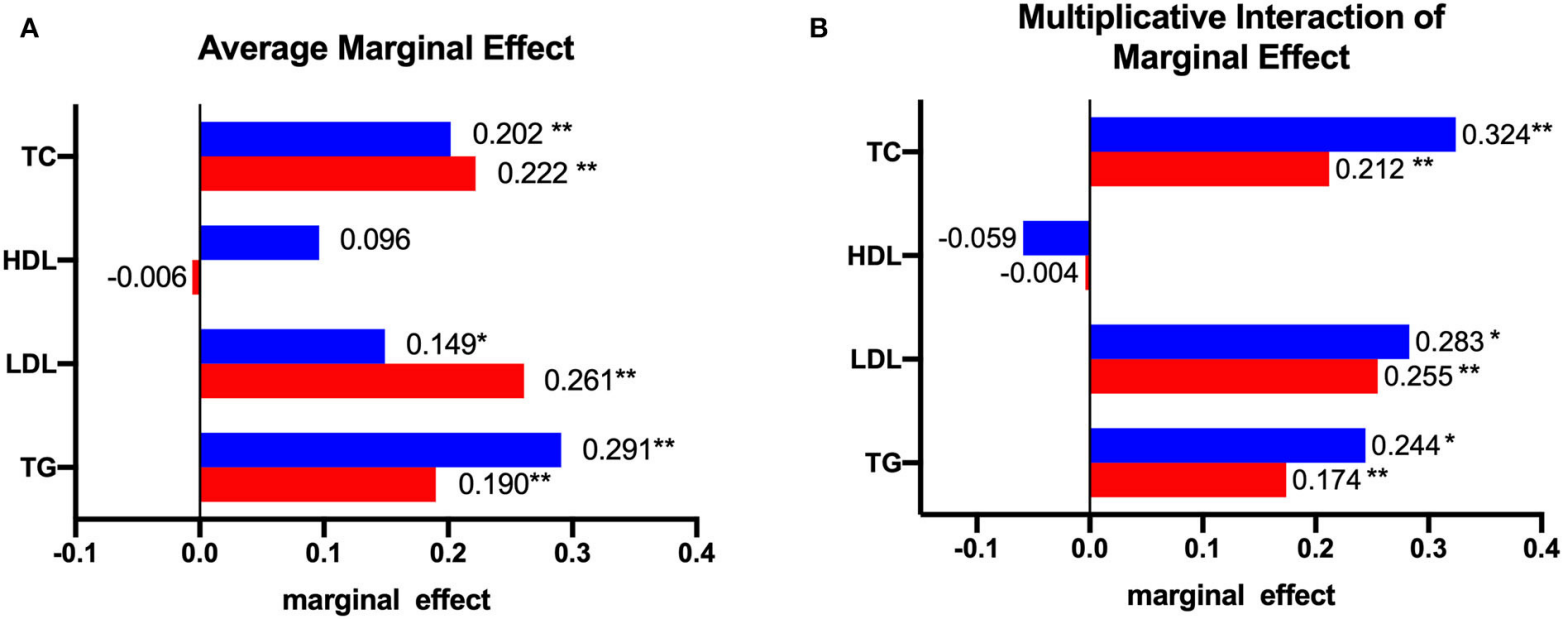
The marginal effect: **(A)** shows the average marginal effect of alcohol consumption and meat consumption on blood lipids, the red bar represents the marginal effect of excessive consumption of meat, and the blue bar represents the marginal effect of alcohol consumption; **(B)** shows the marginal effect of alcohol consumption at a specific value (meat = 1, which means consumed more than 50 g of meat per day), the red bar represents the marginal effect of excessive consumption of meat and no alcohol consumption on blood lipids, and the blue bar represents the effect of excessive consumption of meat and alcohol consumption on blood lipids. *represents *P* < 0.05; **represents *P* < 0.01.

On the basis of logistic regression, we tested the marginal effects of alcohol consumption and excessive meat consumption on blood lipid levels. As shown in Figure 1A, when the means of all other variables in the sample were used, the probability of dyslipidaemia among drinkers was higher than that among non-drinkers (ME = 0.202, *P* < 0.01 for TC; ME = 0.149, *P* < 0.05 for LDL; ME = 0.291, *P* < 0.01 for TG). The incidence of dyslipidaemia among older adults who consumed an excessive amount of meat each day was higher than that among non-drinkers (ME = 0.222, *P* < 0.01 for TC; ME = 0.261, *P* < 0.01 for LDL; ME = 0.190, *P* < 0.01 for TG). The marginal effects of drinking and excessive meat consumption on HDL were not statistically significant. As shown in Figure 1B, the marginal effects of excessive meat consumption but not drinking on the incidence of dyslipidaemia were 0.212 for TC (*P* < 0.01), 0.255 for LDL (*P* < 0.01), and 0.174 for TG (*P* < 0.01). The marginal effects of excessive meat consumption and drinking on the incidence of dyslipidaemia were 0.324 for TC (*P* < 0.01), 0.255 for LDL (*P* < 0.05), and 0.174 for TG (*P* < 0.01). The multiplicative interaction effect of the marginal effects on HDL was not statistically significant.

## Discussion

Diet is the key therapy in the treatment of hyperlipidaemia, and drug therapy should be used only after an adequate trial of diet has failed to achieve satisfactory concentrations of plasma lipids and lipoproteins (41). A high intake of red meat is significantly associated with the prevalence of dyslipidaemia (42). However, meat is an important dietary source of protein. According to the dietary survey data of elderly individuals aged 65 and over, there are three main dietary modes for elderly individuals in China: the “traditional diet” mode, “diversified diet” mode and “animal food” mode (43). The “traditional diet” mode and “animal food” mode can ensure an adequate amount of protein, but the main source of protein is meat. According to a dietary survey of 15 provinces in China, 24.0% of the dietary cholesterol of elderly residents is from red meat (34). Therefore, the control of meat consumption is the key to preventing hyperlipidaemia. According to the survey in this study, 40.6% of the elderly participants eat more meat than the standard. Aging reduces the efficiency of plasma lipoprotein removal mechanisms, and the removal of triglyceride-rich lipoprotein remnants is delayed in elderly individuals (44). This evidence reveals the internal relationship between excessive meat intake and blood lipids among elderly individuals in China.

Alcohol consumption among elderly people increases the risk of hyperlipidaemia. Alcohol metabolism changes with age, and aging organs such as the brain and liver are more sensitive to the toxicity of alcohol (45). Studies have found a link between alcohol consumption and dyslipidaemia risk (46–48). The current study also showed a statistically significant association between drinking behavior and dyslipidaemia. In addition, some studies indicate that drinking more than a certain amount of alcohol increases the risk of dyslipidaemia. A study in Nigeria showed that alcohol consumption above the recommended amount was associated with an almost sixfold increase in the risk of dyslipidaemia (49). A cohort study in China showed that the association between moderate drinking and hyperlipidaemia in middle-aged and elderly women was statistically significant (50). Although this study did not measure alcohol consumption, it counted older adults with an alcohol consumption volume over 14% as drinkers. However, this factor is worth further exploration.

In this study, drinking was not only associated with blood lipids but also had a multiplicative interaction with excessive meat consumption. In fact, some studies have revealed the interaction between alcohol and other substances. Moreover, studies have reported that an interaction between alcohol and specific genes increases blood lipid levels (51, 52). A study showed that the alcohol dietary pattern, characterized by consumption of large amounts of alcoholic beverages, liver, chicken and fish, was associated with the prevalence of dyslipidaemia and its components (25). Another Chinese study showed that alcohol and obesity interact to have an effect on blood lipids (53). Our results are similar to the results of that study. Therefore, although the effect of alcohol on blood lipids is not clear, it is obviously necessary to advocate limiting the amount of alcohol consumed, especially among elderly individuals.

Fish and eggs are considered to be healthier sources of protein. The dietary guidelines for Chinese residents indicate that elderly people need to eat at least 50 g of eggs and 50 g of fish every day. However, only 30.6 and 10.3% of older adults consume more than the recommended amount of fish and eggs, respectively. The Mediterranean diet emphasizes that protein sources other than fruits and vegetables are mainly fish and dairy products. This dietary pattern has the most consistent evidence for efficacy in hypertriglyceridaemia (HTG) (54) and is efficacious for both primary and secondary cardiovascular disease (CVD) prevention (55). However, in this study, the results of logistic regression showed that excessive fish intake was associated with high TG levels. Thus, fish cannot be consumed without restriction simply because it is a healthy source of protein. In this study, the association between excessive consumption of eggs and higher blood lipids was not statistically significant.

Physical activity is a critical component of first-line treatment for elevated blood cholesterol levels, and increasing the amount of physical activity is comparable, superior, or complementary to other healthy lifestyle changes (56). The prevalence of dyslipidaemia has been increasing in the Asia-Pacific region, which is attributed to dietary changes and decreases in physical activity (57). However, in some studies (47), such as this one, the relationship between physical activity and blood lipids was not statistically significant. Another study group found that there was no statistically significant difference in the amount of exercise between the patients with abnormal blood indexes and the healthy subjects before the diagnosis of chronic diseases (58). This may explain to some extent why no relationship was found between physical activity and dyslipidaemia.

### Limitation

China's takeout and snack industries are becoming prevalent. Older adults' diets have many options available in addition to traditional staple foods. However, many foods with a high fat content were not included in this study. Due to the lack of data, edible oils were not included in this study. According to DBI-16 and the results of some studies, the meat of livestock and poultry were not analyzed separately. Furthermore, in this study, the cooking methods of food were not considered either; for example, cooking may be healthier than frying. In addition, this study analyzed only the relationship between alcohol consumption and the risk of dyslipidaemia, did not include the alcohol concentration, and did not define whether alcohol consumption exceeded the standard, although there are standards for excessive alcohol consumption in the *Dietary Guidelines for Chinese residents 2021* (men: ≤ 25 g/day; women: ≤ 15 g/day), which are similar to Swedish and Finnish standards (59). During the statistical analysis, it was found that although the daily alcohol consumption of some respondents did not exceed the standard, all of them exceeded the standard according to the quantity consumed at one time. According to a paper published in *The Lancet*, the level of alcohol consumption that minimized harm across health outcomes was zero (95% UI 0.0–0.8) standard drinks per week (60). Therefore, we adopted the categories “drinker” and “non-drinker” to directly divide the older adults. This study is not an intervention study on diet, so it is impossible to demonstrate any causal relationships between independent variables and blood lipid levels.

## Conclusions

Excessive meat consumption and alcohol consumption increase the risk of hyperlipidaemia in elderly individuals. Alcohol consumption is a regulatory variable that, together with meat consumption, increases the risk of hyperlipidaemia in elderly people, and combined, alcohol and meat consumption increase the risk exponentially. Although our results do not prove that a higher meat intake and alcohol consumption cause hyperlipidaemia, such a relationship is worth exploring from a preventive perspective. This study suggests that it is necessary for elderly individuals to limit their alcohol consumption.

## Data availability statement

The original contributions presented in the study are publicly available. This data can be found here: Figshare, https://figshare.com/, https://10.6084/m9.figshare.19714123.v1.

## Ethics statement

This analysis was approved by the research Ethics Committee of the Tongji University (ref: LL-2016-ZRKX-017). The patients/participants provided their written informed consent to participate in this study.

## Author contributions

XH, HH, JS, ZW, and YW designed the study. XH, HH, and JS conducted the literature analysis. XH, WZ, and ZW conducted the data analysis. HH, WZ, and NC made all tables. NC, YW, and JS produced all figures. JS, ZW, and YW guarantee access to research resources, and all authors participated in the writing, revision, and final review of the manuscript. All authors contributed to the article and approved the submitted version.

## Funding

This study was supported by Shanghai Education Science Research Project (C2021039), National Natural Science Foundation of China (71774116 and 71603182), Shanghai Public Health Outstanding Young Personnel Training Program (GWV-10.2-XD07), the Soft Science Project of Shanghai Science and Technology Commission (22692107200), National Key Research and Development Program of China (2018YFC2000700 and SQ2022YFC3600172), Shanghai Pujiang Program (2020PJC080), and Medical Scientific Research Project of Jiangsu Commission of Health (Z2021020). The funding sources had no role in the design of this study, any role during its execution, analyses, data interpretation, or decision to submit results.

## Conflict of interest

The authors declare that the research was conducted in the absence of any commercial or financial relationships that could be construed as a potential conflict of interest.

## Publisher's note

All claims expressed in this article are solely those of the authors and do not necessarily represent those of their affiliated organizations, or those of the publisher, the editors and the reviewers. Any product that may be evaluated in this article, or claim that may be made by its manufacturer, is not guaranteed or endorsed by the publisher.

## Abbreviations

BMI: body mass index
PA: physical activity
WHO: World Health Organization
TC: total cholesterol
HDL: high-density lipoprotein
LDL: low-density lipoprotein
TG: triglycerides
OR: odds ratios.
